# Supplementary figures and images for: The human amniotic epithelium confers a bias to differentiate toward the neuroectoderm lineage in human embryonic stem cells
Source: eLife. 2022 Jul 11;11:e68035. doi: 10.7554/eLife.68035 (PMC9313526; doi:10.7554/eLife.68035)

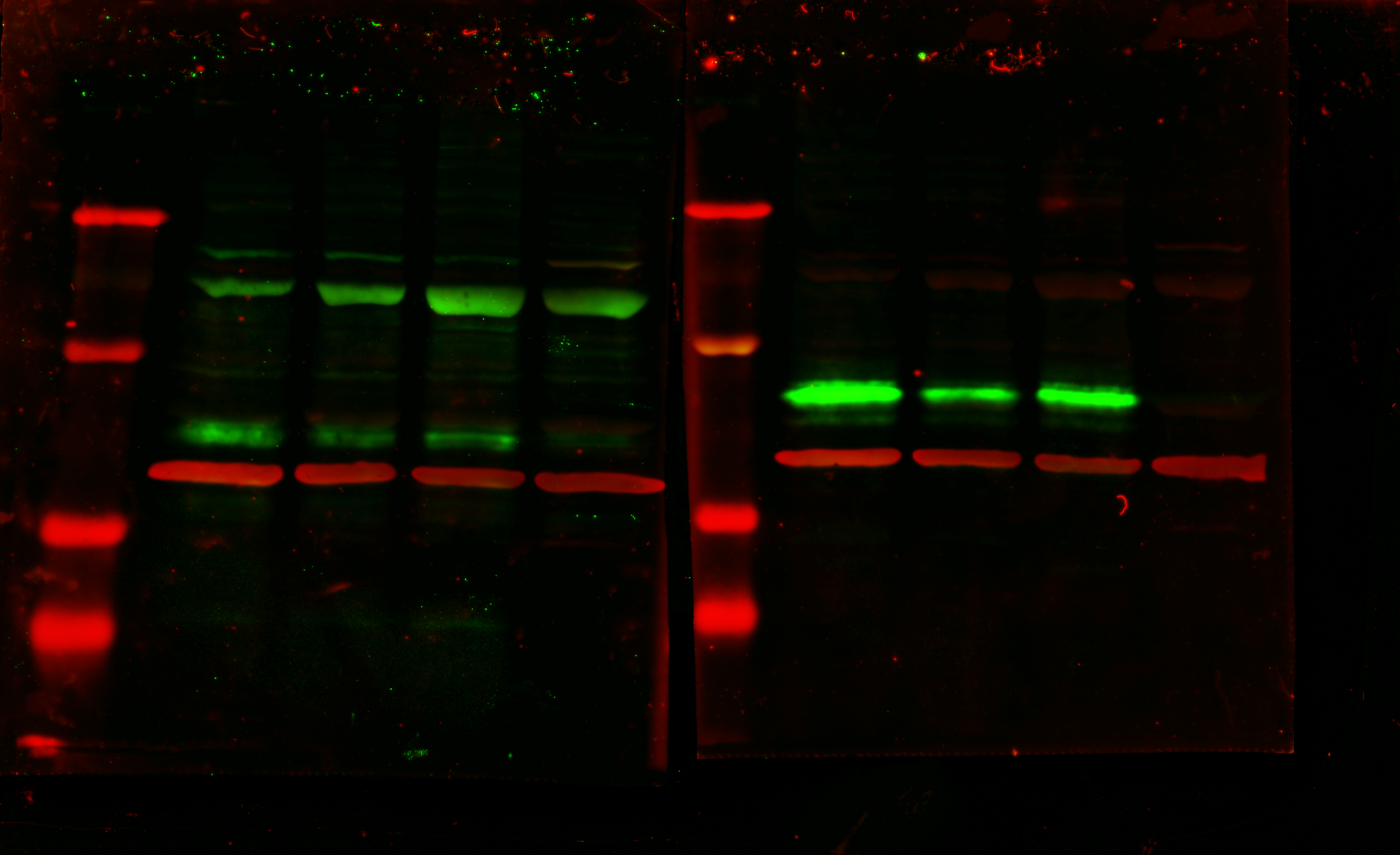

Supplement: Figure 1—source data 1. — Left: replica one of the western blot for NANOG (green, ∼37 kDa) and internal control GAPDH (red, ∼37 kDa). Right: replica one of the western blot for OCT4 (green, ∼42 kDa) and internal control GAPDH (red, ∼37 kDa). The first left lane of each blot corresponds to the molecular weight ladder; second lane, H1 hESC-inactivated mouse embryonic fibroblast (iMEF); third lane, AMIQ hESC-iMEF; fourth lane, AMIQ hESC-human amniotic epithelial cell (hAEC); fifth lane, hAEC. [file elife-68035-fig1-data1.zip › Figure 1-source data 1.tif]

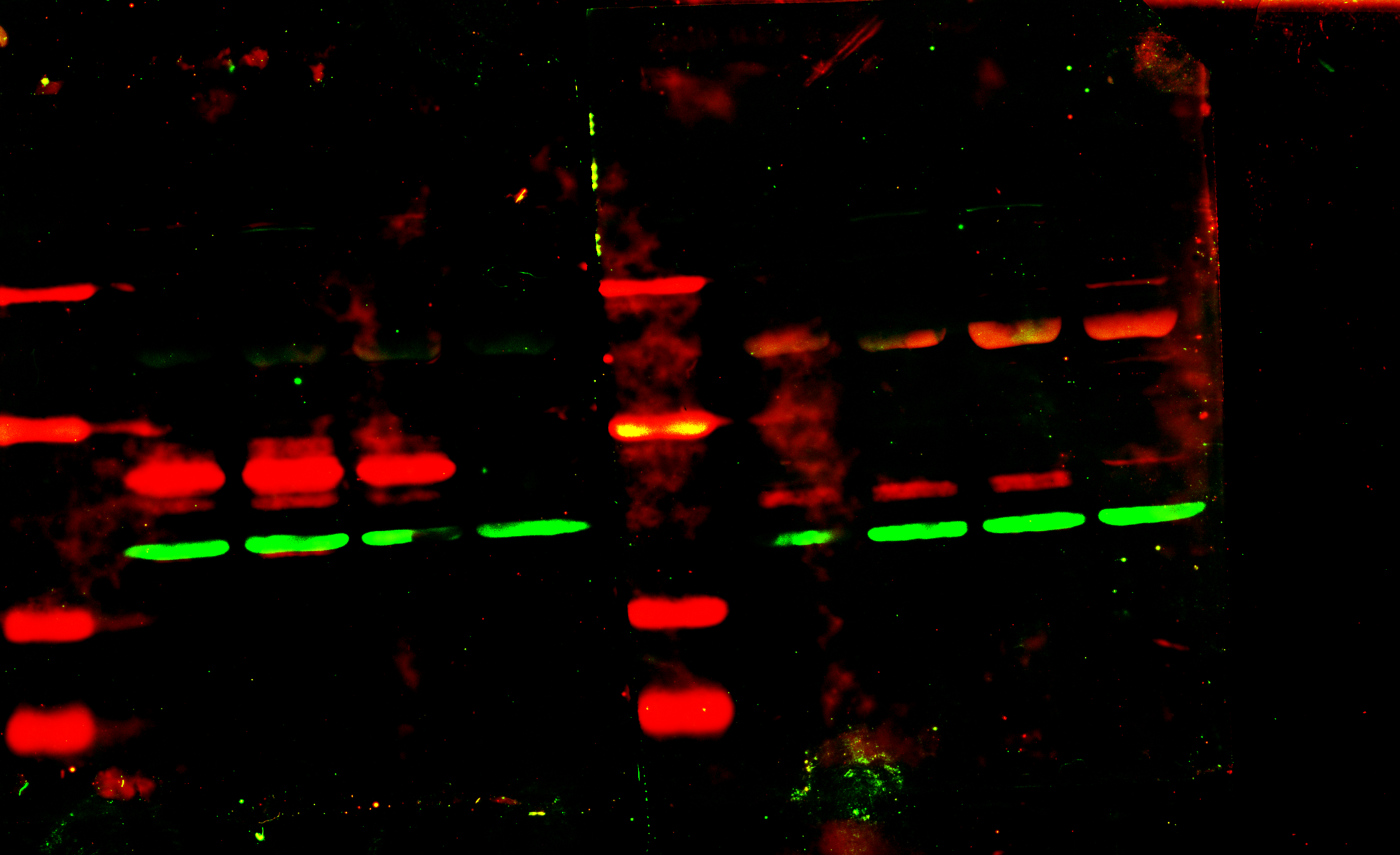

Supplement: Figure 1—source data 2. — Left: replica two of the western blot for OCT4 (red, ∼42 kDa) and internal control GAPDH (green, ∼37 kDa). Right: replica two of the western blot for NANOG (red, ∼37 kDa) and internal control GAPDH (green, ∼37 kDa). The first left lane of each blot corresponds to the molecular weight ladder; second lane, H1 hESC-inactivated mouse embryonic fibroblast (iMEF); third lane, AMIQ hESC-iMEF; fourth lane, AMIQ hESC-human amniotic epithelial cell (hAEC); fifth lane, hAEC. [file elife-68035-fig1-data2.zip › Figure 1-source data 2.tif]

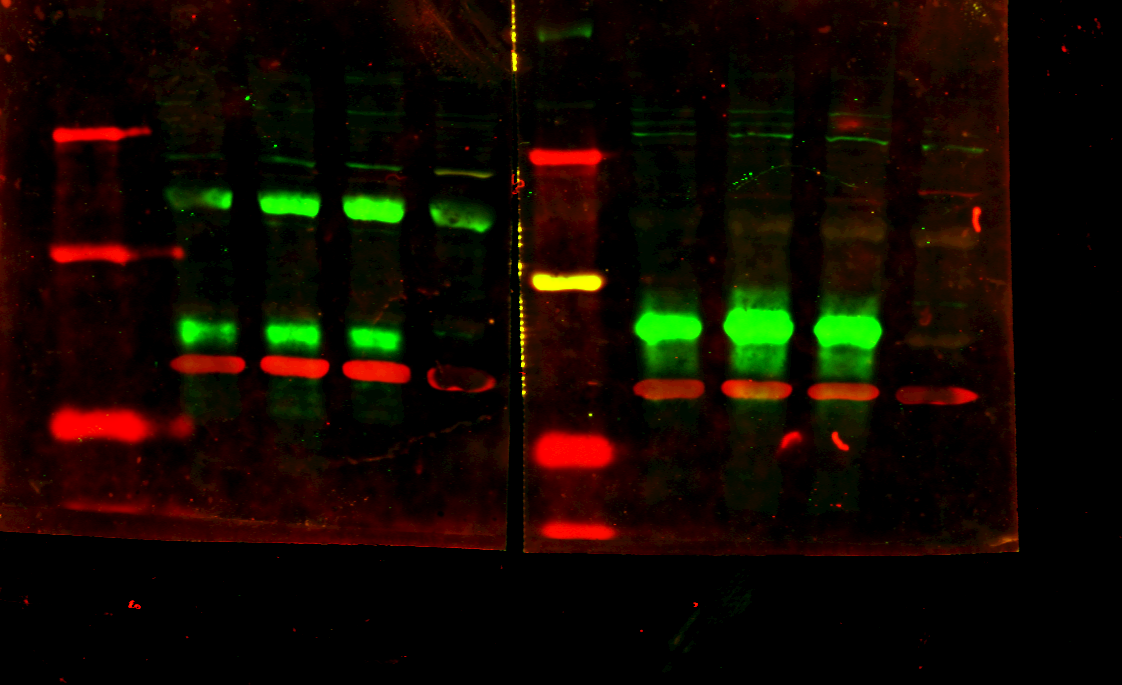

Supplement: Figure 1—source data 3. — Left: replica one of the western blot for NANOG (green, ∼37 kDa) and internal control GAPDH (red, ∼37 kDa). Right: replica one of the western blot for OCT4 (green, ∼42 kDa) and internal control GAPDH (red, ∼37 kDa). The first left lane of each blot corresponds to the molecular weight ladder; second lane, H1 hESC-inactivated mouse embryonic fibroblast (iMEF); third lane, AMIQ hESC-iMEF; fourth lane, AMIQ hESC-human amniotic epithelial cell (hAEC); fifth lane, hAEC. [file elife-68035-fig1-data3.zip › Figure 1-source data 3.tif]

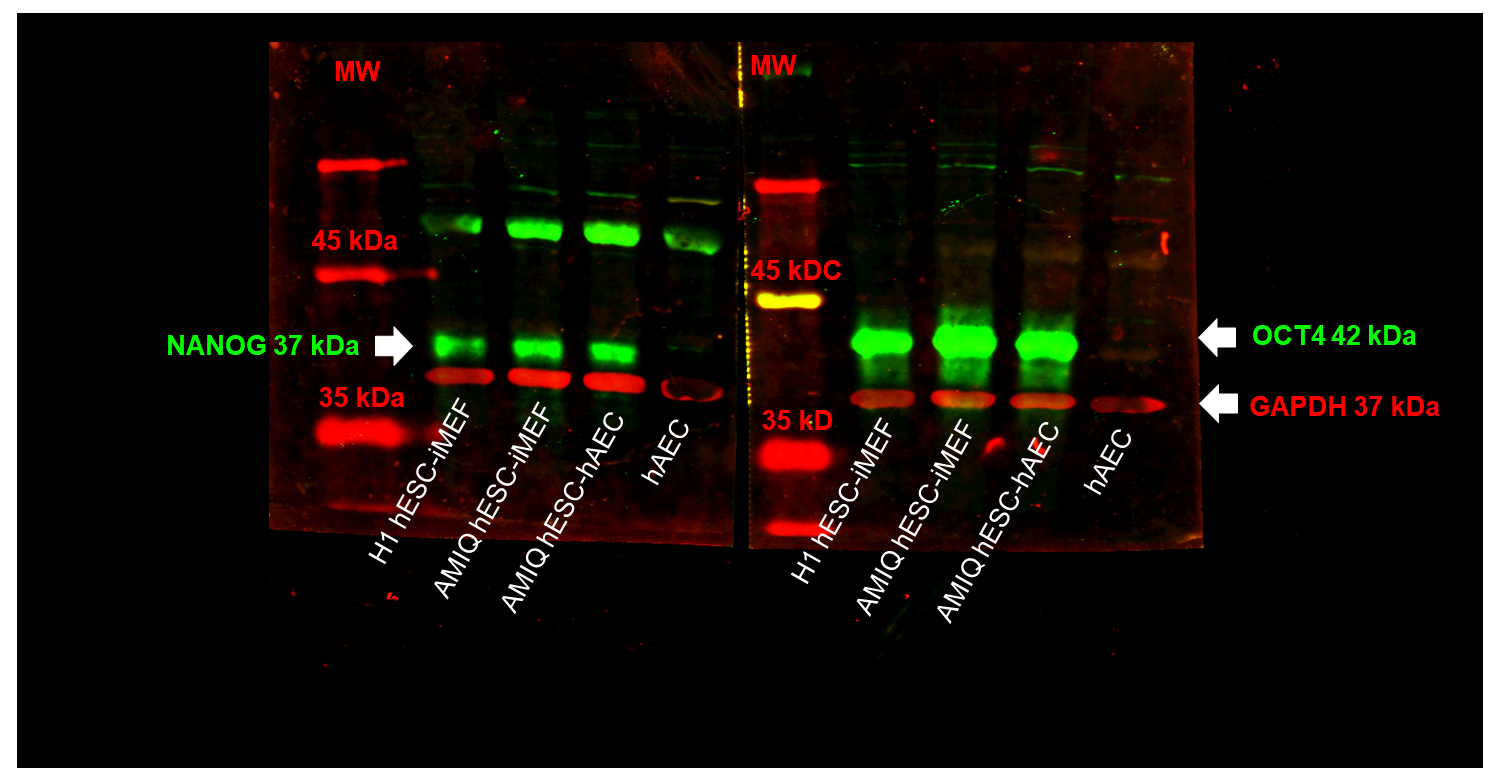

Supplement: Figure 1—source data 4. — Left: representative bands labeled for NANOG (green, ∼37 kDa). Right: representative bands labeled for OCT4 (green, ∼42 kDa) and internal control GAPDH (red, ∼37 kDa). First left lane for each blot, molecular weight ladders (M.W.); second lane, H1 human embryonic stem cell (hESC)-inactivated mouse embryonic fibroblast (iMEF); third lane, AMIQ hESC-iMEF; fourth lane, AMIQ hESC-human amniotic epithelial cell (hAEC); fifth lane, hAEC. [file elife-68035-fig1-data4.zip › Figure 1-source data 4.tif]
